# Supplementary material for: The interactome of intact mitochondria by cross-linking mass spectrometry provides evidence for coexisting respiratory supercomplexes
Source: Mol Cell Proteomics. 2017 Dec 8;17(2):216–32. doi: 10.1074/mcp.RA117.000470 (PMC5795388; doi:10.1074/mcp.RA117.000470)
Supplement: Supplemental Data [file supp_RA117.000470_133922_0_supp_23978_fzffwf.zip › spectra_annotation/mito_spectra_annotation/496-1-1-1-16-1.pdf]

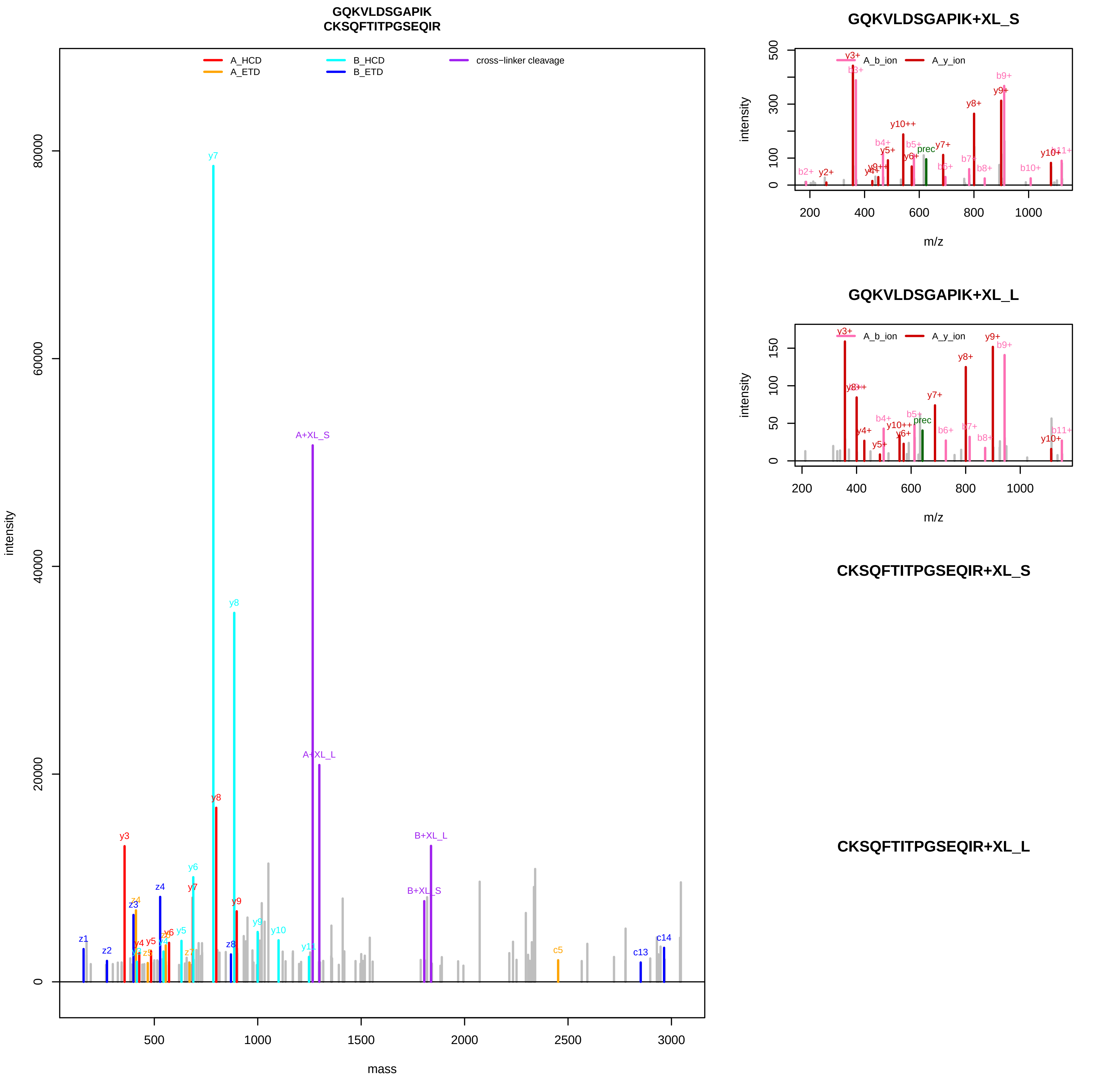
GQKVLDSGAPIK
CKSQFTITPGSEQIR
A_HCD
A_ETD
B_HCD
B_ETD
cross−linker cleavage
y7
A+XL_S
y8
A+XL_L
y8
y3
B+XL_L
B+XL_S
y6
y7
z4
z4
z3
y9
y9
y10
y5
y6
z1
z2
y4
y5
y4
z8
y11
c5
c13
c14
intensity
mass
0
20000
40000
60000
80000
500
1000
1500
2000
2500
3000
GQKVLDSGAPIK+XL_S
A_b_ion
A_y_ion
y3+
b3+
b9+
y9+
y8+
y10++
b4+
y5+
b5+
prec
y7+
y6+
y9++
y4+
b6+
b7+
b8+
b10+
y10+
b11+
b2+
y2+
intensity
m/z
0
100
300
500
200
400
600
800
1000
GQKVLDSGAPIK+XL_L
A_b_ion
A_y_ion
y3+
y9+
b9+
y8+
y8++
y7+
b4+
b5+
prec
y10++
y4+
y6+
b6+
b7+
y5+
b8+
y10+
b11+
intensity
m/z
0
50
100
150
200
400
600
800
1000
CKSQFTITPGSEQIR+XL_S
CKSQFTITPGSEQIR+XL_L
